# A Strategy for Finding the Optimal Scale of Plant Core Collection Based on Monte Carlo Simulation

**DOI:** 10.1155/2014/503473

**Published:** 2014-01-20

**Authors:** Jiancheng Wang, Yajing Guan, Yang Wang, Liwei Zhu, Qitian Wang, Qijuan Hu, Jin Hu

**Affiliations:** ^1^Seed Science Center, College of Agriculture and Biotechnology, Zhejiang University, Hangzhou 310058, China; ^2^Shandong Crop Germplasm Center, Shandong Academy of Agricultural Sciences, Ji'nan 250100, China

## Abstract

Core collection is an ideal resource for genome-wide association studies (GWAS). A subcore collection is a subset of a core collection. A strategy was proposed for finding the optimal sampling percentage on plant subcore collection based on Monte Carlo simulation. A cotton germplasm group of 168 accessions with 20 quantitative traits was used to construct subcore collections. Mixed linear model approach was used to eliminate environment effect and GE (genotype × environment) effect. Least distance stepwise sampling (LDSS) method combining 6 commonly used genetic distances and unweighted pair-group average (UPGMA) cluster method was adopted to construct subcore collections. Homogeneous population assessing method was adopted to assess the validity of 7 evaluating parameters of subcore collection. Monte Carlo simulation was conducted on the sampling percentage, the number of traits, and the evaluating parameters. A new method for “distilling free-form natural laws from experimental data” was adopted to find the best formula to determine the optimal sampling percentages. The results showed that coincidence rate of range (CR) was the most valid evaluating parameter and was suitable to serve as a threshold to find the optimal sampling percentage. The principal component analysis showed that subcore collections constructed by the optimal sampling percentages calculated by present strategy were well representative.

## 1. Introduction

Genome-wide association studies (GWAS) have been successful in identifying genes in quantitative traits at an unprecedented rate [1–3]. GWAS proved a way to investigate the relationship between molecular genetic variation and variation in quantitative traits. Comparing the traditional linkage mapping method, GWAS have much higher resolution because they involve studying a natural population rather than the offspring of crosses, and associations in natural populations are typically on a much finer scale because they reflect historical recombination events [4]. However, GWAS have largely not been applied in plants. This is due mainly to the lack of resources like those seen in other well-developed systems, such as the human genome HapMap project [5]. A system contains maximum genetic diversity of quantitative traits with minimum repetitiveness will promote GWAS in plants. Therefore, core collection may be an ideal resource for GWAS in plants. A core collection is a representative sample of the whole collection with minimum repetitiveness and maximum genetic diversity of a plant species [6]. The core collection serves as a working collection that can be evaluated and utilized preferentially, which saves large funds and provides a convenient way to study germplasm resources and find useful genes [7–12].

The main aim of core collection research is to find effective methods to conserve maximum genetic diversity by minimum accessions. One common approach for constructing a core collection is grouping the whole collection by growing regions or ecotype, then selecting representative core accessions from each group to form subcore collections, and combining all subcore collections to form the final core collection [13, 14]. Most core collection researches focused on finding efficient ways in core accessions selection [15–17]. However, there is not a widely accepted method for constructing a core collection up to now. One major reason is those too many effect factors in representativeness of a core collection, such as sampling percentage, data type, number of traits observed, genetic diversity of plant germplasm, grouping method, and sampling method [13, 16, 18].

It is well known that with the sampling percentage increasing, the representativeness of a core collection increased. However, it is not a widely accepted core population scale, especially in core collection constructed based on data of quantitative traits. The observed values of quantitative traits are more easily affected by environment than those of qualitative traits. More traits accumulate more environmental errors and experimental errors, which leads to less representativeness of core collections. Therefore, it is necessary to find a method to eliminate environmental and experimental errors of data observed from quantitative traits in core collection construction. Many researchers just set a fixed sampling percentage in core collection construction [19, 20]. It might lead to the loss of genetic diversity. Many germplasm collections are large scale and over 10,000 accessions conserved, which means that only 1% difference of the sampling percentage will lead to over 100 accessions “in or out” of the core collection. It sometimes takes risk. We have investigated the effect of the scale of quantitative trait data on the representativeness of core collection in the former research [14]. However, the system for determining the optimal sampling percentage of a core collection has not been established. The objective of this research was to use subcore collections as working material to develop a strategy to determine the optimal sampling percentage on plant core collection based on Monte Carlo simulation. The strategy helps to establish a germplasm system with more accurate and representative core collection for GWAS in plants.

## 2. Materials and Methods

### 2.1. Materials

168 Liaoning local cotton varieties were selected from the whole genebank and planted in the experimental farm of Liaoning Economy Crop Research Institute (Liaoning, China) for 2 years with 2 replications per year. There were 6 rows and 80 columns in each replication. The observed data of twenty quantitative traits were recorded. There were 11 agronomy traits (plant height, height of fruit branch, length of fruiting node, length of boll stalk, number of fruiting branch per plant, bolls per plant, incidence of infected plant, index of wilt disease, growth period, boll weight, and lint percentage), 5 fiber traits (length, uniformity, strength, elongation, and micronaire), and 4 seed traits (seed length, seed width, ratio of length to width, and kernel weight). The same dataset has been used and published in 2013 [14]. The year, row, and column effects were treated as the fixed effects, and the genotypic effect was treated as the random effect.

### 2.2. Genetic Model

The observed values of any quantitative trait could be expressed as

(1) $$\begin{array}{c} Y_{hk(ij)}=\mu +E_{h}+R_{i(h)}+C_{j(h)}+G_{k(ij)}+GE_{hk(ij)}+\varepsilon_{hk(ij)}, \end{array}$$

where *μ* is the population mean; *E*_*h*_ is the fixed effect of the hth environment; *R*_*i*(*h*)_ is the fixed effect of the *i*th row within the *h*th environment; *C*_*j*(*h*)_ is the fixed effect of the *j*th column within the *h*th environment; *G*_*k*(*ij*)_ is the random effect of the *k*th genotype within the *i*th row and the *j*th column, *G*_*k*(*ij*)_ ~ (0, *σ*_*G*_^2^); *GE*_*hk*(*ij*)_ is the random effect of the interaction between the *h*th environment and the *k*th genotype, *GE*_*hk*(*ij*)_ ~ (0, *σ*_*GE*_^2^); and *ε*_*hk*(*ij*)_ is the residual effect, *ε*_*hk*(*ij*)_ ~ (0, *σ*_*ε*_^2^) [21]. The minimum norm quadratic unbiased estimation (MINQUE) method was adopted to calculate the variance components [21]. To unbiasedly predict the genotypic values of the 168 cotton varieties, the adjusted unbiased prediction (AUP) method was used because it gives more accurate prediction of variance for predicted genetic effects than the best linear unbiased prediction (BLUP) method [21].

Mixed linear model approach was used to predict genotypic values of accessions to eliminate environment effect, row effect, column effect, GE (genotype × environment) effect, and residual effect [21]. Core collections constructed by predict genotypic values are more precise and representative than by observed values [22, 23].

### 2.3. Method for Subcore Collection Construction

Least distance stepwise sampling (LDSS) method [22] was adopted to construct subcore collections. This method performs sampling based on the subgroup with least genetic distance, which could efficiently eliminate redundant accessions and ignore the effect of different cluster methods to the final subcore collection. The process is as follows. First, the genetic distances between accessions are calculated. Then, one accession from a subgroup with the least genetic distance is randomly sampled and another is removed. Next, genetic distances among the remained accessions are calculated again, and the sampling is performed by the same way. The stepwise samplings are performed until the percentage of the remained accessions reaches the given sampling percentage and the subcore collection is achieved.

### 2.4. Selection of Evaluating Parameters and Genetic Distances for Subcore Collection

In order to determine the precise sampling percentage, a sensitive and effective evaluating parameter is needed. Seven evaluating parameters for data of quantitative trait were served as checking options. These were mean difference percentage (MD), variance difference percentage (VD), changeable rate of maximum (CR_max_), changeable rate of minimum (CR_min_), changeable rate of mean (CR_mea_), coincidence rate of range (CR), and variable rate of coefficient of variation (VR). These parameters are formulated as follows [24]:

(2) $$\begin{array}{c} \text{MD}=\left(\frac{S_{t}}{n}\right)\times 100\%, \end{array}$$

where *S*_*t*_ is the number of traits which have significant difference (*α* = 0.05) of means between the initial germplasm group and subcore collection and *n* is the total number of traits;

(3) $$\begin{array}{c} \text{VD}=\left(\frac{S_{F}}{n}\right)\times 100\%, \end{array}$$

where *S*_*F*_ is the number of traits which have significant difference (*α* = 0.05) of variances between the initial germplasm group and subcore collection and *n* is the total number of traits;

(4) $$\begin{array}{c} \text{CR}=\frac{1}{n}\overset{n}{\underset{i=1}{\sum }}\frac{R_{C(i)}}{R_{I(i)}}\times 100, \end{array}$$

where *R*_*C*(*i*)_ is the range of the *i*th trait of subcore collection, *R*_*I*(*i*)_ is the range of the corresponding trait of the initial germplasm group, and *n* is the total number of traits;

(5) $$\begin{array}{c} \text{VR}=\frac{1}{n}\overset{n}{\underset{i=1}{\sum }}\frac{{\text{CV}}_{C(i)}}{{\text{CV}}_{I(i)}}\times 100, \end{array}$$

where CV_*C*(*i*)_ is the coefficient of variation of the *i*th trait of subcore collection, CV_*I*(*i*)_ is the coefficient of variation of the corresponding trait of the initial germplasm group, and *n* is the total number of traits;

(6) $$\begin{array}{c} {\text{CR}}_{\max}=\frac{1}{n}\overset{n}{\underset{i=1}{\sum }}\frac{{\mathrm{Max}}_{C(i)}}{{\mathrm{Max}}_{I(i)}}\times 100, \end{array}$$

where *Max*_*C*(*i*)_ is the maximum value of the *i*th trait of subcore collection, *Max*_*I*(*i*)_ is the maximum value of the *i*th trait of the initial germplasm group, and *n* is the total number of traits;

(7) $$\begin{array}{c} {\text{CR}}_{\min}=\frac{1}{n}\overset{n}{\underset{i=1}{\sum }}\frac{{\mathrm{Min}}_{I(i)}}{{\mathrm{Min}}_{C(i)}}\times 100, \end{array}$$

where *Min*_*C*(*i*)_ is the minimum value of the *i*th trait of subcore collection, *Min*_*I*(*i*)_ is the minimum value of the *i*th trait of the initial germplasm group, and *n* is the total number of traits;

(8) $$\begin{array}{c} {\text{CR}}_{\text{mea}}=\frac{1}{n}\overset{n}{\underset{i=1}{\sum }}\frac{{\text{Mea}}_{C(i)}}{{\text{Mea}}_{I(i)}}\times 100, \end{array}$$

where Mea_*C*(*i*)_ is the mean value of the *i*th trait of subcore collection, Mea_*I*(*i*)_ is the mean value of the *i*th trait of the initial germplasm group, and *n* is the total number of traits.

The calculation on evaluating parameters was based on core accessions selected from nonstandardized group after subcore collections were constructed based on standardized group. Six commonly used genetic distances (Euclidean distance, Euclid; standardized Euclidean distance, Seuclid; Mahalanobis distance, Mahal; city block distance, Cityblock; cosine distance, Cosine; and correlation distance, Correlation) combining unweighted pair-group average (UPGMA) cluster method were used to construct subcore collections [25]. In each genetic distance, 84 sub-core collections were constructed from the sampling percentage of 10% to 30% with 4 replications. All the 7 evaluating parameters were calculated in each combination (a sampling percentage plus a replication). To investigate the validity of the evaluating parameters, homogeneous population assessing method was adopted. Significance of difference for the same evaluating parameter at different sampling percentage was tested by variance analysis. Tukey's test (*α* = 0.05) was used to perform multiple comparison and letter marking method was used to show the comparing results. The number of homogeneous populations of Tukey's test (e.g., according to alphabetical order, if the largest letter was “c,” the homogeneous populations were 3; if the largest letter was “f”, the homogeneous populations were 6) was used to assess the validity of each evaluating parameter. Larger number of homogeneous populations meant more subcore collections being distinguished, and the corresponding evaluating parameter was more valid [24].

### 2.5. Method for Determining the Optimal Sampling Percentage Based on Monte Carlo Simulation

The sampling percentage and the number of traits were set as two changing factors. With a selected genetic distance, subcore collections were constructed from the sampling percentage of 10% to 30% (sampling percentages under 10% were too small to calculate evaluation parameters because the initial population just contained 168 accessions) in each number of traits. Meanwhile, in each sampling percentage, subcore collections were constructed from the number of traits of 1 to 20. Selected evaluating parameters were calculated in each subcore collection. The upper procedure was replicated 20 times, and the trait order was randomized in each replication to homogenize trait effect (different trait contained different extent of variation). The mean values of an evaluating parameter of all replications were calculated in each combination (a sampling percentage × a number of traits). The simulation results generated a matrix of the mean values of a selected evaluating parameter.

Based on the upper data, a new method for “distilling free-form natural laws from experimental data” [26–28] was adopted to find a reasonable formula on the relationship between the sampling percentage, the number of traits, and the value of a selected evaluating parameter. Subsequently, the equation for the relationship between the sampling percentage and the corresponding number of traits was achieved by setting a reasonable value of a selected evaluating parameter. The optimal and precise sampling percentage could be achieved from that equation.

### 2.6. Data Management

Tukey tests were performed using ANOVA procedure in SAS software (version 6.11) [29]. Procedure for finding the reasonable formula was performed by Eureqa software (version 0.83) (http://creativemachines.cornell.edu/eureqa). Other data processing was conducted by MATLAB software (version 6.5) [30].

## 3. Results

### 3.1. The Validity of 7 Evaluating Parameters and 6 Genetic Distances

Euclid, Seuclid, and Cityblock generated far more total homogeneous populations than other genetic distances. However, there was only one homogeneous population generated by Euclid and Cityblock in VD (Table 1). VR had most homogeneous populations in Euclid, Seuclid, Mahal, and Cityblock while had only one in Cosine and Correlation (Table 1). CR had the most homogeneous populations in Cosine and the second largest number of those in Euclid, Seuclid, Mahal and Cityblock (Table 1). MD, VD, VR, CR_min_, and CR_mea_ had only one homogeneous population in Correlation, and those in CR and CR_max_ were 2 and 4, respectively (Table 1). By this way, the validity of the 7 evaluating parameters could be sorted as CR, VR > CR_max_, CR_mea_, CR_min_ > MD, and VD. Since VR showed too bad representation in Cosine and Correlation, considering the general purpose, Seuclid genetic distance and the evaluating parameter of CR were selected.

### 3.2. Finding the Formula for the Relationship between the Sampling Percentage, the Number of Traits, and the Value of CR

**Figure 1:**
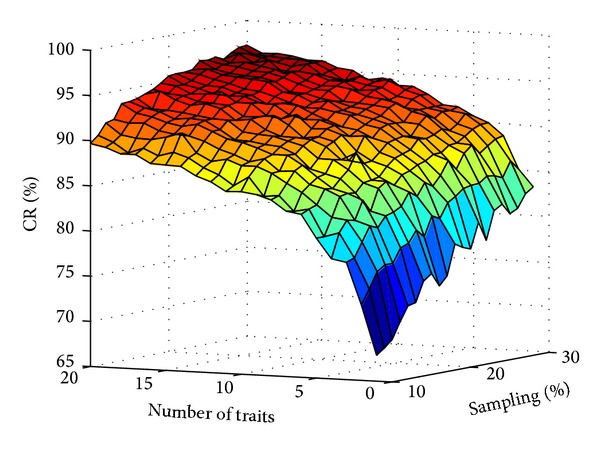
The 3D curved surface of CR changing by the sampling percentage and the number of traits.

Data matrix based on the simulation results produced a curved surface in three dimensions (the sampling percentage, the number of traits, and the value of CR) (Figure 1). Both the sampling percentage and the number of traits affected the value of CR (Figure 1). In a similar way to logarithmic tendency, the value of CR increased dramatically when the number of traits and the sampling percentage were small, while it increased smoothly with those two factors reaching high level (Figure 1). Further analysis was needed for finding the internal laws in that changing system.

**Figure 2:**
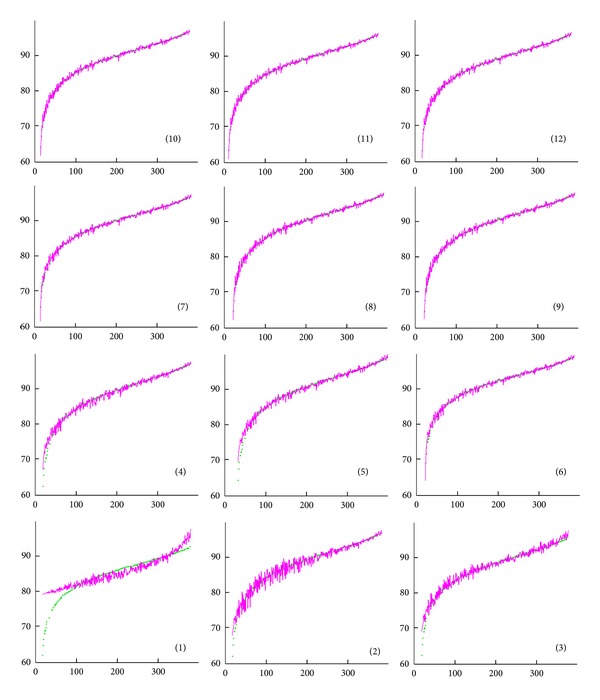
The fitness of the 12 formulas. The number on *x*-axis was the index of the validation data. The number on *y*-axis was the value of the validation data. The dots showed the validation data, and the fold line showed the solution based on the selected formula. The numbers in parentheses were the formula number.

By means of the method mentioned above, several formulas were distilled by Eureqa based on the simulation results of CR. Formulas with the *R* ^2^ (the coefficient of determination) lower than 0.7000 were ignored. Therefore, 12 formulas were summarized and sorted by the *R* ^2^ in Table 2.  Figure 2 showed the fitness of the selected formula on the validation data (the data matrix based on the simulation results). The validity of the formula was also determined by the complexity (“size”) and the accuracy (“error”) of the validation data. Formulas (1), (2), and (3) were not available because of the high error and low *R* ^2^ (Table 2). Formulas (9), (10), (11), and (12) showed low error and high *R* ^2^ but too large size (Table 2). Formula (6) showed lower error and higher *R* ^2^ than (4) and (5) and showed slightly higher error and slightly lower *R* ^2^ than (7) and (8) (Table 2). Formula (6) showed more fitness than (4) and (5) and showed similar fitness to (7) and (8) (Figure 2). Considering the size, (6) was selected.

In general, CR needs to be not less than 80% in a reasonable subcore collection [6, 23, 31]. When CR was set to be 80.00 (percentage), (6) was transformed to the following equation:

(9) $$\begin{array}{c} \text{percentage}=\frac{e^{2.75}+5.09\times \text{Traits}+4.24}{\text{Traits}}. \end{array}$$

**Figure 3:**
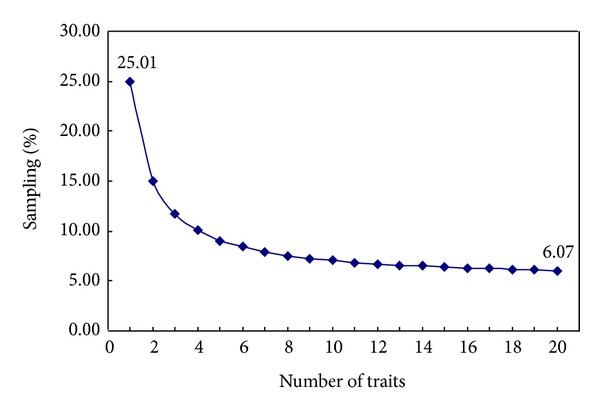
The relation curve of the sampling percentage and the number of traits when CR's value was set to 80%. 25.01 and 6.07 were the optimal sampling percentage (%) when the number of traits was 1 and 20, respectively.

The optimal sampling percentage per number of traits was calculated based on the upper equation. The optimal sampling percentage decreased from 25.01% to 6.07% with the number of traits increasing from 1 to 20 (Figure 3).

### 3.3. Validation of the Optimal Sampling Percentage

To make full use of genetic diversity and eliminate trait effect, values of all the 20 traits were used as working data. Subcore collections constructed by LDSS method based on Seuclid distance combining UPGMA cluster method were used to investigate the validity of different sampling percentage (treat). To prove the validity of the upper subcore collections, completely random selected populations were served as controls (CK). At the three sampling percentages of 6.07% (the optimal one calculated by the upper equation when the number of traits was set to 20), 10.00%, and 15.00%, the treats showed much higher CR and VR than CKs (Table 3). At the sampling percentage of 6.07%, the treat's CR was higher than 80% (Table 3). In the treats, with the sampling percentage increasing, CR increased, VR decreased, and the other three parameters did not change much (Table 3).

**Figure 4:**
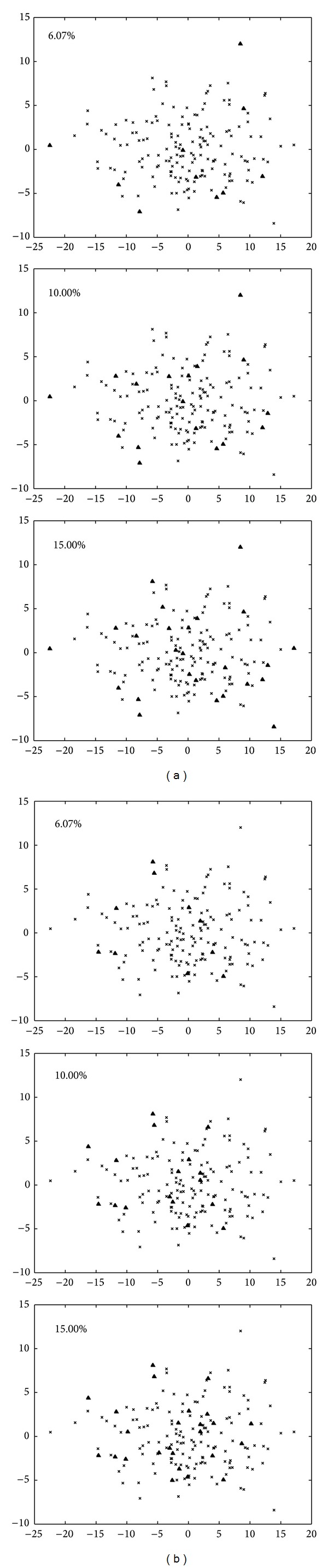
Principal component plots of core accessions and reserve accessions in the sampling percentages of 6.07%, 10%, and 15%. The axes represented the first two principal components. The upward pointing triangles represented the core accessions; the crosses represented the reserved accessions. The left column showed plots for subcore collection constructed by LDSS method based on Seuclid distance combining UPGMA cluster method (treat); the right column showed plots for subcore collection constructed by complete random selection (CK).

The principal component analysis was conducted to validate subcore collections constructed by the upper three sampling percentages. Principal component plots of core accessions and reserve accessions at the three sampling percentages were drawn in Figure 4. The first two principal components represented 76.43% genetic variation of the total. Compared to the CK, core accessions of treat showed more symmetrical distribution in the whole germplasm group at the sampling percentage of 6.07%, and most extreme accessions were selected (Figure 4). Treat showed well representative at the sampling percentage of 6.07% and showed more representative at the sampling percentages of 10.00% and 15.00% (Figure 4).

## 4. Discussion

The first key for a rational sampling percentage is preserving genetic diversity as far as possible, and the second one is reducing the collection size. Therefore, some parameters for evaluating genetic diversity preservation in core collection are needed. For data of quantitative trait, homogeneous population assessing method was adopted in present research and CR was selected as the working parameter. CR relates to the percent of range of traits preserving in core collection, as a more intuitionistic evaluating parameter; CR is suitable for the evaluation of core collection [6, 23, 31]. Larger CR means more representativeness of a core collection [22, 25]. For data of qualitative trait or molecular marker, the Shannon-Weaver Diversity Index (SDI) was suggested as a valid evaluating parameter by some researchers [32–34].

The sampling percentage of a core collection has long been under debate. Brown [35] suggested a sampling percentage of 5%~10%. Yonezawa et al. [36] thought 20%~30% of the sampling percentage was needed to well conserve the genetic diversity of the whole germplasm collection. In very large collections, even 1% approximately of the sampling percentage was suggested (minicore) by some researchers [33, 37–39]. Logozzo et al. [40] constructed a common bean core collection with over 55% of the sampling percentage. In general, most core collection sizes are 10%~30% of the initial collection [15, 19, 41]. In our opinion, a perfect ratio or fixed size for all core collections does not exist, and different plant or different constructing goal needs different sampling percentage.

The “Eureqa” method was first suggested to identify and document analytical laws that underlie physical phenomena in nature [26]. The method can automatically search a serious of solutions to explain the changing system. In present research, the sampling percentage, the number of traits, and the value of CR composed a changing system. The *R* ^2^ showed that the selected formula (6) distilled by the “Eureqa” method could well explain the laws of the three factors of the sampling percentage, the number of traits, and the value of CR. The 3D figure showed that the three factors might be logarithmic relationship. The subsequent selected formula clearly presented logarithmic laws in expression, which prove the guess. There were also some formulas that showed lower error and higher *R* ^2^ than the best formula selected in present research. However, the sizes of those formulas were too large, which meant that they were too complex to use in practice. There is another thing that needed to be paid attention to that is the factors in the formula have their own value ranges. Setting values out of range in the formula will produce odd results. The present strategy is large computational cost, because it is composed of mixed linear model, LDSS method, Monte Carlo simulation, and “Eureqa” method. The main factor for determining the computation time is the accession number in the initial collection. A big size collection makes the computational difficulty when the present strategy is used. Since a core collection is constituted by subcore collections, we resolved the difficulty by conducting our strategy within the domain of subcore collection. The optimal sampling percentage of a core collection will be achieved by combining all the computational results of subcore collections.

## Figures and Tables

**Table 1:** The number of homogeneous populations of Tukey's test (*α* = 0.05) of 7 evaluating parameters in each germplasm population from the sampling percentage of 10% to 30%.

Parameter	Genetic distance					
	Euclid	Seuclid	Mahal	Cityblock	Cosine	Correlation
MD	3	4	2	3	1	1
VD	1	3	3	1	1	1
CR	8	7	5	8	6	2
VR	15	11	6	12	1	1
CR_max_	6	5	4	5	6	4
CR_min_	3	3	3	3	3	1
CR_mea_	3	5	3	2	1	1

Total	39	38	26	34	19	11

**Table 2:** The formulas distilled by Eureqa based on the simulation results of CR.

Size^a^	Formula	Error^b^	*R* ^2^ ^c^	FN^d^
34	$f(x,y)=81.00+0.11y+2.63\log(xy)-\frac{62.06y+472.15}{x+xy+2.63\log(xy)}$	0.075	0.9935	(12)
28	$f(x,y)=78.65+0.09y+3.01\log(xy)-\frac{60.77y+345.54}{x+y+xy}$	0.075	0.9934	(11)
26	$f(x,y)=77.94+0.09y+3.10\log(xy)-\frac{53.22y+323.37}{x+y}$	0.075	0.9934	(10)
24	$f(x,y)=70.56+0.09y+4.38\log(x+xy)-\frac{29.63y+310.22}{x+xy}$	0.076	0.9932	(9)
20	$f(x,y)=92.17+4.16\log(y)-\frac{291.40}{1.34+x+2.90\log(y)}$	0.078	0.9926	(8)
18	$f(x,y)=69.88+\frac{y-61.33}{x}+4.56\log(xy-8.75)$	0.083	0.9917	(7)
15	*f*(*x*, *y*) = 65.90 + 5.12log(*xy* − 5.07*y* − 4.24)	0.090	0.9896	(6)
13	*f*(*x*, *y*) = 64.26 + 5.39log(*xy* − 5.02*y*)	0.114	0.9757	(5)
12	$f(x,y)=85.72+5.40\log(y)-\frac{133.68}{x}$	0.120	0.9746	(4)
11	*f*(*x*, *y*) = 70.37 + 0.40*x* + 5.46log(*xy*)	0.161	0.9619	(3)
9	*f*(*x*, *y*) = 59.55 + 5.95log(*xy*)	0.180	0.9571	(2)
7	*f*(*x*, *y*) = 84.24 + 0.03*xy*	0.389	0.7222	(1)

^a^The complexity of the formula; ^b^the error of the fitted formula; ^c^ *R* ^2^: the coefficient of determination; ^d^FN: formula number.

**Table 3:** The values of five evaluating parameters in subcore collections constructed by three sampling percentages with 20 traits.

Subcore collection	Sampling percentage	Parameter				
		CR	VR	CR_max_	CR_min_	CR_mea_
Treat^a^	6.07%	83.46	167.13	95.85	97.49	97.54
	10.00%	89.84	152.09	97.55	97.89	99.30
	15.00%	94.88	140.39	98.90	99.53	99.15

CK^b^	6.07%	48.91	95.62	92.50	85.35	100.52
	10.00%	56.00	94.51	94.86	85.75	101.05
	15.00%	61.49	94.62	95.36	87.64	100.36

^a^Subcore collection constructed by LDSS method based on Seuclid distance combining UPGMA cluster method; ^b^subcore collection constructed by complete random selection.
